# Presence of Soybean Vein Necrosis Orthotospovirus (Tospoviridae: Orthotospovirus) in Pakistan, Pakistani Scientists’ and Farmers’ Perception of Disease Dynamics and Management, and Policy Recommendations to Improve Soybean Production

**DOI:** 10.3390/v17030315

**Published:** 2025-02-25

**Authors:** Asifa Hameed, Cristina Rosa, Paige Castillanos, Edwin G. Rajotte

**Affiliations:** 1 Department of Entomology, College of Ag Sciences, The Pennsylvania State University, University Park, PA 16802, USA; asifa_hameed_sheikh@yahoo.com; 2 Department of Plant Pathology and Environmental Microbiology, College of Ag Sciences, The Pennsylvania State University, University Park, PA 16802, USA; czr2@psu.edu; 3 Office of International Programs, College of Ag Sciences, The Pennsylvania State University, University Park, PA 16802, USA; pxd171@psu.edu

**Keywords:** SVNV, qRT-PCR, ELISA, policy, survey

## Abstract

Soybean vein necrosis orthotospovirus (SVNV: Tospoviridae: Orthotospovirus) is a well-recognized thrips-vectored and seed-borne virus common in the United States (U.S.), Canada, and Egypt. Pakistan started the commercial cultivation of soybeans in the 1970s, when some soybean cultivars were imported from the U.S. to meet the country’s domestic requirement of oil, poultry, animal feed, and forage. A survey of farmers and scientists was conducted in the Punjab and Khyber Pakhtunkhwa provinces of Pakistan to understand perceptions of SVNV in the indigenous Pakistani community. Concurrently, soybean fields were sampled for SVNV presence at the National Agricultural Research Institute in Islamabad, Pakistan. Based upon survey and SVNV detection results through ELISA and qRT-PCR, a policy was developed. Overall, we found that SVNV was present in Islamabad, Pakistan in USDA-approved soybean cultivars. Although scientists knew about general thrips biology and insecticides, knowledge about identification of vectors (Thrips species) was not significantly different between the scientists and the farmers. Scientists at the Islamabad location were more aware of crop production technology and pests. This study reports that Pakistan needs to strengthen its research institutes, scientists’ and farmers’ capacity building, and extension programs to understand the disease complex in soybean crops.

## 1. Introduction

Soybean is one of most valuable crops for oil seed, food, forage, biodiesel, feed, and leguminous nitrogen fixing, improving soil structure through nitrogen fixation and enhancing farmer income along with multiple other benefits [1,2,3,4]. Soybean is the main crop in Brazil and Argentina and the second most important broad-acre agricultural crop in the United States, providing high cash benefits to farmers [5,6]. Brazil, Argentina, and the USA combined produce 85% of the world’s soybeans, while China, Pakistan, and several other countries are importers of this valuable commodity [7].

Soybean is affected by a plethora of diseases caused by bacteria, fungi, and viruses as well as insects and mites [8,9]. Their effects on soybean plants result in reductions in yield. On average, due to these diseases, about 60.66 USD are lost per acre in the USA and Canada [10]. In 2008, a new pathogen, soybean vein necrosis orthotospovirus (SVNV), belonging to the family Tospoviridae, order Bunyavirales, was first identified in Tennessee, USA [11,12,13]. SVNV is the first Tospoviridae member which has been described as seed-transmissible [14]. Soon after its discovery, it was found in 2012 in Canada and in 2017 in Egypt [15,16,17]. Moreover, 17 plant species belonging to seven families can act as hosts of the virus either in field or laboratory conditions [18]. Among these are mung beans, which are part of the diet of people in southeast Asia. Six species of thrips can facilitate transmission of the virus inoculum to alternative host plants and have been identified as potential virus vectors [16,17,19].

Thrips are important pests in soybean and can cause up to a six percent reduction in yield. In addition, heavily infested plants may even die [20,21]. In the United States, the virus is naturally transmitted by *Neohydatothrips variabilis* (Beach, 1896); however, two other thrips species, viz., *Frankliniella tritici* (Fitch, 1855) and *F. fusca* (Hinds, 1902), can also transmit the virus [19,22,23,24,25,26]. In Egypt, *Megalurothrips sjostedti* (Tribom, 1908), *Caliothrips phaseoli* (Hood, 1912), and *F. occidentalis* (Pergande, 1895) were also identified as potential virus vectors, although the natural infection vector remained *N. variabilis* [17].

Pakistan is a significantly agro-based country. Of the total labor force of Pakistan, 45% of people are engaged in agriculture, and about 70% of the Pakistani population lives in rural areas [27]. The agricultural share of Pakistani exports is 80%. Pakistani agricultural exports consist of cotton products, rice, leather, garments, and hosiery products. For a poor country like Pakistan, it is important to develop strategies to cut back imports [28].

Pakistan is a populous country (fifth largest in the world), where about 33% people (55 million) live below the poverty line [28]. The current human development index in Pakistan is 0.562, which is slightly lower than in Bangladesh (0.65). The human development index is an indicator of the education level, literacy rate, life span, and per capita income. In countries where the literacy rate is higher, life span is longer, and countries where the GDP/GNP (gross domestic product or gross national product) is higher receive a higher ranking. The poor HDI of Pakistan reflects its poverty, illiteracy, and the poor overall health of Pakistani populations.

Pakistan shares a long border with India, China, Iran, and Afghanistan. Pakistan has fertile agricultural land and is an exporter of diversified agricultural products including cotton, mango, and corn. Most Pakistani people live in villages and speak the Pakistani national language, Urdu, although there is a wide range of native languages in different provinces. Most of the population consists of small-scale subsistence farmers who rely on the production of their crop for livelihood.

Pakistan consists of four different provinces, viz., Punjab, Sindh, Balochistan, and Khyber Pakhtunkhwa Province or KPK. Although soybean can be grown in all provinces, only in KPK Province do a few farmers currently grow soybeans. In fact, during this survey, we could find 50 farmers who grew soybean only in KPK. Farmers in Punjab and Sindh mostly grow other crops. Soybean, as a spring crop, is planted at the beginning of the year, with a seed rate range of 80–100 kg/ha. Soybeans are planted with a seed drill at 30–45 cm row spacing in spring and autumn. The distance between plants is maintained at 3–5 cm. Fertilizers are applied at an N:P:K ratio of 25:65:50 kg/ha. The number of times the field is irrigated is variable, with irrigation performed six times in spring and three to four times in the autumn crop.

Although Pakistan is an agricultural country, it still imports soybean oil and seed to meet the needs of livestock, poultry, and aquaculture. In fact, Pakistan spends about 55.047 billion Pakistani rupees for the import of soy products [29].These soybean seeds, meals, and oil are mainly purchased from the United States [30]. During the last two decades, there has been a tremendous increase in Pakistan’s imports of soybean. In 1985, about 150 tons of soymeal and 200 tons of soybean seed were imported, but in 2019, this reached 2 million metric tons, costing 106.48 billion Pakistan rupees [30]. A country where socio-economic factors constrain the population, resulting in poor education and health and lower lquality of life, this expenditure could be used to alleviate poverty, increase employment, enhance the education level, and enhance the quality of life To reduce reliance on imports and enhance Pakistani’s own domestic oil production, the soybean cultivars Lee and BARC were imported from Ohio State University in 1965–1968 [31,32,33]. From this germplasm, Pakistan developed its own soybean cultivars through cooperation with and support from the USDA, and Pakistan started a self-sufficiency program to promote soybean production in the 1970s.

The soybean cultivars proved well suited to the warm climate of Pakistan. The cultivars developed were NARC-I, NARC-II, Williams-82, Rawal-1, Rawal-2, Ajmeri, Faisal-I, and Faisal-II [32,34]. The Pakistan Agriculture Research Centre developed new projects to encourage farmers to grow soybeans using a rotation scheme of cotton–soybean–cotton, rice–soybean–rice, and wheat–soybean–wheat. Soybean days were celebrated, and farmers were invited to observe the crop production technologies, but the crop production was not improved. In fact, the soybean in general was not warmly accepted in the Pakistani farming community. The adoption of soybean in the Pakistani farming community could not meet its target, and the cultivation of soybean in the areas of Balochistan and in the fertile lands of Punjab remained minimal [30,31,32,33] until finally declining to zero in 2011. KPK Province, where area is measured not in hectares but in kanals (one hectare is equal to 20 kanals), is the only province where a few farmers grow soybeans. There might be multiple reasons behind the poor adoption of the soybeans in the Pakistani farming community. Overall, new crop adoption starts with close cooperation between extension agents, scientists, and farmers [32,34]. The purpose of this study was to determine whether SVNV is present in Pakistan, as Pakistani soybean cultivars derive from cultivars imported from the U.S., where the virus is present. Furthermore, we wanted to discover whether the farming/scientist community has noticed any symptoms resembling the ones caused by SVNV. This study had the following objectives:

- We analyzed the farmers’ and scientists’ perceptions of soybean cultivation to identify reasons behind the low adoption of soybean.
- We posed questions about SVNV dispersal, vectors, quarantine, and management to Pakistani farmers and scientists. The overall purpose of the research was to determine what the scientists and farmers know about SVNV, whether they can identify thrips and SVNV, and whether they practice biological and chemical control.
- We also analyzed gaps in the knowledge of the Pakistani farming community regarding soybean cultivation and the management practices they are adopting.

Overall, this study is important because:

- This is the first survey article linking farmers’ and scientists’ views about the poor soybean industry in Punjab and KPK in Pakistan.
- This is the first study describing SVNV presence in soybean plants on USDA-approved Pakistani soybean cultivars.
- This is the first study to develop a policy portfolio for the enhanced production of soybeans in Pakistan, considering the survey results.
- This information would also be useful for evolutionary pathologists and integrated pest management scientists to understand dispersal of SVNV in importer countries.

## 2. Materials and Methods

### 2.1. Survey of SVNV in Soybean Cultivars Mantained at the National Agricultural Research Centre (NARC), Islamabad

During August 2019, fields of soybeans at the National Agricultural Research Centre (NARC), Islamabad, were examined for vein necrosis and chlorosis. Six symptomatic plants were selected, and one leaf per plant was subjected to DAS (double antibody sandwich) ELISA specifically for soybean vein necrosis virus, according to the protocol of the manufacturer (Agdia Incorporation, Elkhart, IN, USA; Cat #SRA4320) and as modified by Hameed et al. [21], at the National Institute of Genomics and Advanced Biotechnology, National Agricultural Research Institute, Islamabad. Virus-positive controls from Agdia, non-symptomatic plants, and PBST buffer were also included in the experiment. The wells showing 3-fold higher O.D. value than the negative control at 450 nm were considered to contain samples that were virus-positive.

Since two of these plants tested positive for SVNV, we extended the virus survey and collected 140 leaves from symptomatic and asymptomatic plants of seven varieties, viz., Ajmeri, Faisal P-17, NARC-16, NARC-2, Rawal-1, SA7260, and Williams-98 (20 leaves per variety). These leaves were also subjected to DAS-ELISA as described above.

### 2.2. Detection of Virus Through qRT-PCR

To confirm ELISA detection, qRT-PCR was performed on a subset of soybean plants that differed from the ones subjected to ELISA but were collected in the same week. Each plant was treated as a biological replicate. In brief, RNA was extracted from three non-symptomatic and three symptomatic plants using the Purelink RNA Mini kit (Cat #12183018A, Life Technologies, Invitrogen BioServices India Pvt. Ltd., Bengaluru, India), following the manufacturer’s protocol. The detailed procedure is described by Hameed et al. [21]. RNA quality and quantity were checked through nanodrop (BioSpec-nano UV–Vis spectrophotometer, Shimadzu, Kyoto, Japan). The RNA was immediately stored at −70 °C.

RNA was also extracted from thrips (field-collected through paint brush and beating sheet method as described above with a few modifications). About 20 thrips were used for RNA extraction, and 3 RNA extractions were performed to obtain 3 biological replicates. Instead of 600 µL lysis buffer, 50 µL of buffer was used. Final elution of RNA was done with 30 µL of DNA- and RNA-free water. The detailed procedure is described by Hameed et al. [21].

### 2.3. cDNA Synthesis

cDNA was synthesized from RNA using a Thermo Scientific RevertAid First Strand cDNA Synthesis Kit (Thermo Scientific, Invitrogen BioServices India Pvt. Ltd., Bengaluru, India). A master mix was prepared by adding 1 µL random hexamer primers, 4 µL 5× reaction buffer, 1 µL ribonuclease inhibitor, 2 µL 10 mM dNTP mix, and 1 µL RevertAid M-MuLV RT (200 U/µL) and RNA for a total volume of 20 µL. The reaction conditions were: incubation at 50 °C followed by cDNA synthesis at 42 °C for 60 min, with the reaction terminated by heating for 5 min at 70 °C. A detailed procedure is described by Hameed et al. [21].

### 2.4. Real-Time PCR Quantification of the Virus in the Infected Aamples

qRT-PCR was performed using Maxima SYBR Green/ROX qPCR Master Mix (Thermo Scientific, India, Catalogue #K0221) in an ABI StepOne real-time PCR machine (Applied Biosystem, Carlsbad, CA, USA). For an internal control factor, *Glycine max* elongation factor (GmELF1-b) (GenBank accession number: XM_003545405) primer was used (Table S1). SVNV-NP (nucleocapsid protein) and SVNV-NSs (non-structural silencing protein) were used (Keough et al., 2016). qPCR conditions were 95 °C (2 min) followed by 40 cycles of 95 °C (10 s), then 55 °C (30 s), and a final melt curve (65 °C to 95 °C), increased by 0.5 °C each 5 s. The detailed procedure is described by Hameed et al. [21].

### 2.5. Farmer Attitude Survey

**Figure 1:**
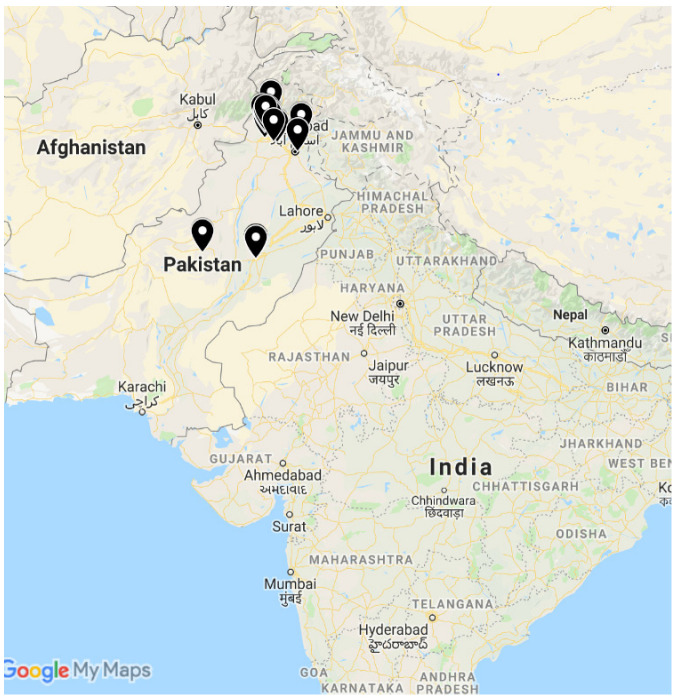
Survey locations in Pakistan during 2019. The survey was conducted in Multan, Punjab, Pakistan; Islamabad, Punjab, Pakistan; and KPK, Pakistan. A total of 50 farmers and 73 scientists participated in the interview.

A total of 50 farmers (from Khyber Pakhtunkhwa, Pakistan) and 73 scientists (23 scientists from Multan and 50 scientists from Islamabad, Punjab, Pakistan) were selected during June–September 2019 (Figure 1). KPK Province and Islamabad, Punjab are closer to one another than either is to southern Punjab (Multan). The selection of scientists was a random process and included scientists working on different crops in research institutes at Islamabad and Multan locations. However, all farmers from a list obtained from the Oil Seed Research Institute, NARC, Islamabad, were included in the survey. This was because the principal investigator could not find any soybean farmers in Punjab, so all KPK soy growers (50 farmers) were interviewed. The farmers were interviewed individually. The questions asked were both open-ended and closed questions. Some farmers were interviewed in person, while for distant farmers (4 total), the questionnaire was sent through mail. In order for the farmers and scientists to completely understand the SVNV disease, color photographs were provided with each question. Photographs of the virus-infected plants were developed and videos taken in scientists’ research labs. Different questions were posed to the farmers than to the scientists about SVNV symptoms, vectors, and dispersal (Table S1). The principal investigator received prior IRB training (IRB Study #00012337) and approval from the PSU Office of Research Protections to conduct the survey in Pakistan. The questions were asked in the local Pakistani Urdu language.

### 2.6. Statistical Analysis

For the SVNV field survey, pairwise multiple comparison through Tukey HSD at a 5% level of significance was performed.

For the survey questions which had binary answers (i.e., yes or no, spray or do not spray, whether resistant varieties should be developed or not), the analysis was conducted according to binomial modeling in R version 3.5.3. The answers were coded (0 = No, 1 = Yes), and closed-ended questions were analyzed through binomial logistic regression and post hoc test to determine significance, probability, and standard error. For the open-ended questions, we used the Chi-square test and the Chi-square post hoc test to determine *p*-values and the difference between the variables. Rates of responses to each questionnaire were calculated. Scientists working on different crops and at different research institutes and geographical locations were surveyed. Thus, the overall awareness of scientists and farmers regarding SVNV was analyzed.

Graphs were built through R version 3.5.3. Labeling of graphs was done through Adobe Photoshop version 19.1.5.

## 3. Results

### 3.1. Detection of SVNV in Plants and Insects Through ELISA and Incidence of the Virus in Pakistani Soybean Cultivars

**Figure 2:**
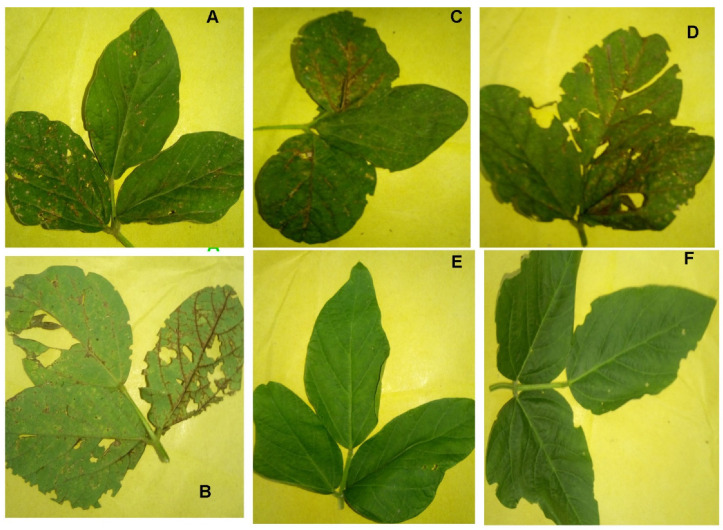
Symptomatic infected leaf samples taken from soybean field at NARC, Islamabad, Pakistan. (**A**) Minor necrosis of the leaf. (**B**) Necrotic veins present on other side of leaf as well, as the disease progresses. (**C**) As disease progresses, both alternate leaves are infected. (**D**) All the leaflets of the soybean leaf showing symptoms of necrosis. (**E**,**F**) Healthy plant leaves collected from the field.

**Figure 3:**
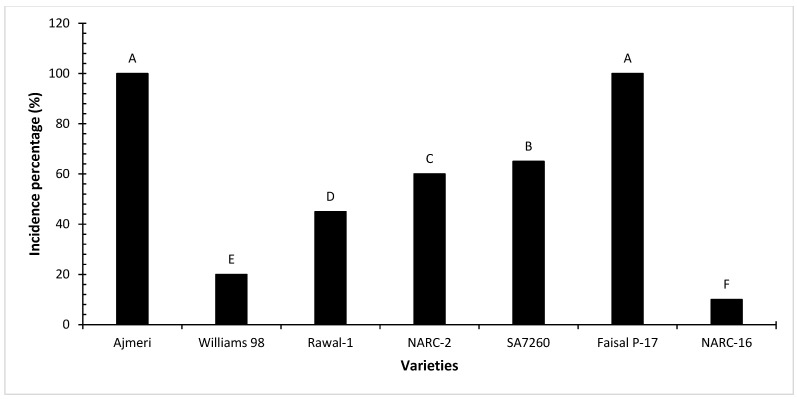
Incidence of soybean vein necrosis virus in USDA-approved Pakistani soybean cultivars. Here, the letters A–F represent the statistical ranks obtained after pairwise multiple comparison through Tukey HSD at a 5% level of significance.

Out of the initial six tested leaves, two symptomatic leaves were positive and four were negative. Figure 2 represents some of uninfected and SVNV-positive leaves found in Pakistan. The incidence percentage of the virus in USDA-approved soybean cultivars ranged from 10 to 100%. Ajmeri and Faisal P-17 had 100% incidence, while NARC-II and SA7260 had 60 and 65% incidence, respectively. Rawal-1, Williams-98, and NARC-16 had 45%, 20%, and 10% SVNV incidence, respectively (Figure 3; Table S3).

qRT-PCR results confirmed that the virus was present in Pakistan, as all three tested symptomatic plants and the three groups of thrips tested positive (Table S2).

### 3.2. Scientists’ and Farmers’ Responses

In general, there was little soybean experience among survey respondents, reflecting the unfulfilled potential of soybean production in Pakistan.

#### 3.2.1. Number of Scientists Interviewed

A total of 73 scientists were surveyed, including 23 scientists from Multan and 50 scientists from Islamabad. The scientists’ responses demonstrated their knowledge of the soybean crop, production technology, soybeans’ pests, and their management. Overall, 21 scientists were working on soybeans in the National Agricultural Research Center in Islamabad, Pakistan. Only two graduates with PhDs in agricultural plant breeding specialization were found in the southern Punjab conducting research on soybeans (Table 1).

#### 3.2.2. Landholding Capacity of Pakistani Soybean Farmers and Soybean Cultivar Preference

**Figure 4:**
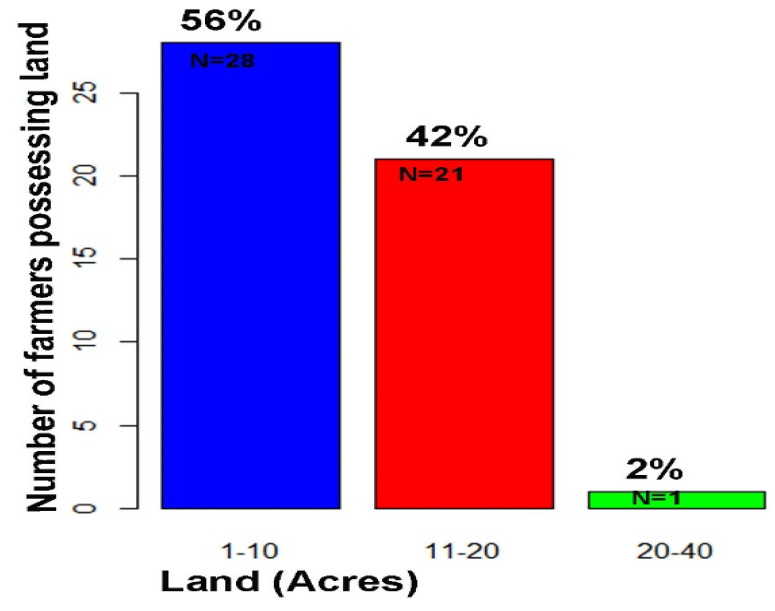
Landholding capacity of Pakistani farmers in KPK, Pakistan.

**Figure 5:**
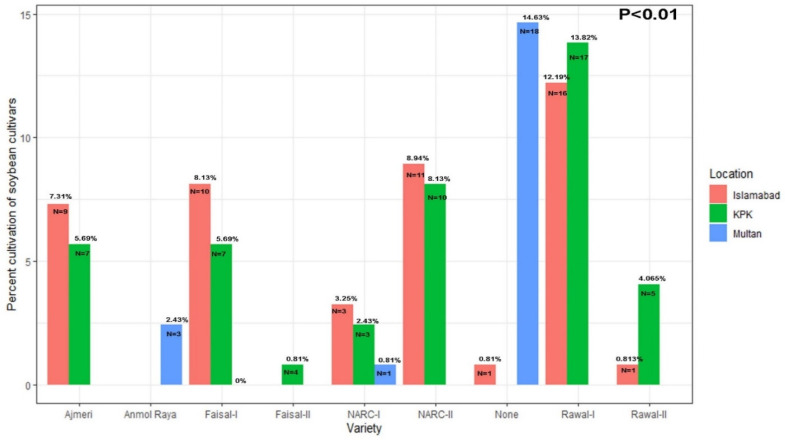
Based upon survey results, the soybean cultivars’ cultivation frequency in Pakistan.

In our survey data set, only one farmer had more than 20 acres of land, while a majority of them held below 20 acres (χ^2^ = 69.68, *p* < 0.01) (Figure 4). Fifty-six (56) percent of 50 farmers (28 total) had 1–10 acres of land, about 42% (21 farmers) had 11–20 acres of the agricultural land, and 2% of 50 farmers (1 farmer) had more than 20 acres of agricultural land (Figure 5).

When asked about the soybean cultivars planted, most scientists reported that they do not work on soybean crops (Figure 5). In Multan, 19 scientists (14.63%) reported that they had never cultivated soybeans on their farms (Figure 5). In Islamabad, the rate of cultivation of soybean cultivars was higher than in Multan. Only one scientist (0.813%) in Islamabad reported never having cultivated soybeans. Three scientists (2.43%) in the southern Punjab (Multan location) responded that they recognized Anmol Raya as a soybean cultivar, when it is in fact a rapeseed cultivar (*p* < 0.01) (Figure 5). Anmol Raya was considered a soybean crop by three scientists (2.43%) in Multan (Figure 5), although Anmol Raya is a rapeseed and not a soybean. In KPK, all farmers grew soybeans.

**Figure 6:**
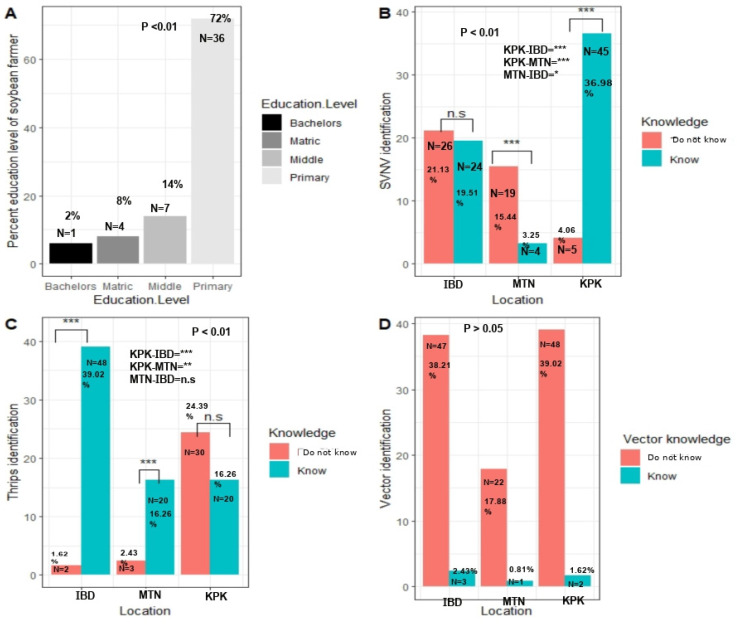
Responses of farmers and scientists to different questions. (**A**) Education level percentages of the farmers. (**B**) Knowledge of SVNV in scientists and farmers. Farmers and scientists were given colored photographs, and upon seeing symptoms, the farmers recognized similar symptomatic plants they have observed in their fields. (**C**) Ability to identify thrips among other insects. The comparison was done on the basis of the Chi-square test and binomial logistic regression. (**D**) Vector knowledge in scientists and farmers. Overall, the scientists are aware of thrips in general. KPK, IBD, and MTN correspond to Khyber Pakhtunkhwa Province, Pakistan; Islamabad, Punjab, Pakistan; and Multan, Punjab, Pakistan. Here KPK-IBD means comparison of responses between farmers at location KPK and Islamabad. KPK-MTN means comparison of responses between farmers at location KPK and Multan. MTN-IBD means comparison of responses between scientists at location Multan and Islamabad. Here * represent significant difference below *p* < 0.05, while ** means comparison of responses at 5% level of significance showed *p* < 0.01, while *** represents highly significant difference among mean responses at location IBD, MTN and KPK.

Nine scientists (7.32%) in Islamabad grew the Ajmeri variety. No scientist in Multan had grown the Ajmeri variety (Figure 6). Seven farmers (5.69%) in KPK grew the Ajmeri variety (Figure 5). Anmol Raya was considered a soybean crop by three scientists (2.43%) in Multan (Figure 5), although Anmol Raya is a rapeseed and not a soybean. This showed a lack of detailed knowledge about the soybean crop in southern Punjab, even among scientists. Faisal-I was cultivated by ten scientists (8.13%) in Islamabad, seven farmers (5.69%) in KPK, and zero scientists (0.0%) in Multan (Figure 5). NARC-I was cultivated by four scientists (3.25%) in Islamabad, three farmers (2.439%) in KPK, and zero farmers (0.0%) in Multan (Figure 5). NARC-II was cultivated by eleven scientists (8.94%) in Islamabad, ten farmers (8.13%) in KPK, and zero scientists (0.0%) in Multan. NARC-II was cultivated by eleven scientists (8.94%) in Islamabad, ten farmers (8.13%) in KPK, and zero scientists (0.0%) in Multan (Figure 5). One scientist in Islamabad (0.813%), zero farmers in KPK, and eighteen scientists (14.63%) in Multan had never seen soybean before (Figure 5). Total respondents from Multan were 23, which shows how meager the knowledge the scientists had about the soybean crop in southern Punjab. Rawal-I was cultivated by sixteen scientists (12.19%) in Islamabad, 17 farmers (13.82%) in KPK, and zero scientists in Multan. Rawal-II was cultivated by one scientist (0.81%) in Islamabad location, five farmers (4.06%) in KPK, and zero scientists (0.0%) in Multan (Figure 6).

### 3.3. Education Level and Knowledge of SVNV and Vector Thrips

In response to questions about education level, we found significant differences in the education of farmers (χ^2^ =119.2, *p* < 0.01) (Figure 6A). About 72% of 50 farmers (36 total) were only primary-qualified, which is regarded as a fifth-grade education. Fourteen percent (seven farmers) were eighth-grade qualified, and 8% (four farmers) were tenth-grade qualified. Only 6% (three total) were graduates (Figure 6A). A person qualified for 14 grades of schooling is considered a graduate. This can be equivalent to a bachelor of arts, bachelor of sciences, or any specific field, e.g., engineering.

Almost all scientists were able to distinguish thrips from all other organisms, which meant that the scientists overall recognized the thrips (*p* > 0.05) (Figure 6C). Specifically, 48 scientists in Islamabad, 20 farmers in KPK, and 20 scientists in Multan (39.024, 16.26, 16.26%) were able to identify thrips among many other insects (Figure 6B). Only two scientists in Islamabad (1.62%), thirty farmers (24.390%) in KPK, and three scientists (2.43%) in Multan were not able to identify thrips.

The identification of SVNV-symptomatic plants was significantly variable in all locations (χ^2^ = 31.645, *p* < 0.01) (Figure 6C,). Twenty-six scientists in Islamabad, five farmers in KPK, and nineteen scientists in Multan (21.13, 4.06, 15.44%) reported that they have never seen SVNV-symptomatic plants in the field. Twenty-four scientists in Islamabad, forty-five farmers in KPK, and four scientists in Multan (19.51, 36.58, 3.25%) reported that they had seen similar symptomatic plants in the soybean fields (Figure 6C). Identification of vector species was non-significantly different at different locations (*p* > 0.05) (Figure 6D).

### 3.4. Insecticide Application by Farmers

**Figure 7:**
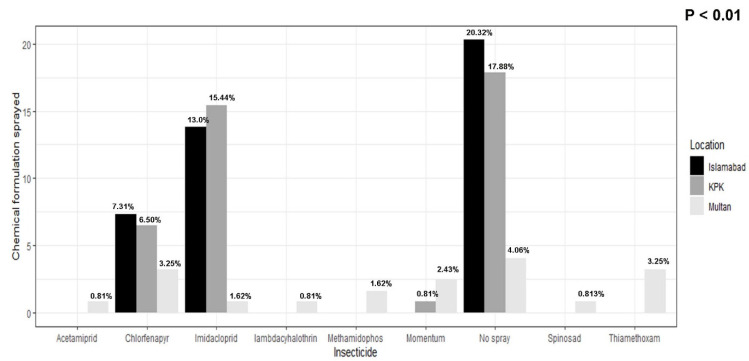
Most-used insecticide by farmers and scientists. The value on the y-axis is the percentage of respondents, and on x-axis are the commercial insecticides which farmers described using on their crops to control pests. Some farmers also reported that they do not use pesticide, so the portion of scientists who do not use pesticide is also mentioned herein.

Asked what insecticide they sprayed in their soybean fields, most of the farmers and scientists selected the no-spray option, followed by imidacloprid (*p* < 0.05, χ^2^ = 45.101) (Figure 7). One scientist (0.81%) in Multan described that he used acetamiprid to control thrips on several crops (Figure 7). In KPK and Islamabad, no farmer or scientist reported spraying acetamiprid. Thiamethoxam was used by four scientists (3.25%) to control thrips population (Figure 4, Figure 5, Figure 6 and Figure 7). Eight scientists (7.31%), nine farmers (6.50%), and four scientists (3.25%) in Islamabad, KPK, and Multan, respectively, reported that they sprayed chlorfenapyr to control thrips populations. Sixteen scientists (13.0%) in Islamabad, nineteen farmers (15.44%) in KPK, and two scientists (1.62%) in Multan reported that they sprayed imidacloprid to control thrips population (Figure 7). One scientist (0.81%) in Multan reported using the lambda cyhalothrin to control thrips population. Two scientists (1.62%) in Multan described spraying methamidophos to control thrips population (Figure 5). Three scientists (1.62%) sprayed momentum to control thrips population. Twenty-five scientists, twenty-two farmers, and six scientists in Islamabad, KPK, and Multan, respectively (20.325, 17.886, and 4.065%), reported that they never sprayed soybeans (Figure 7). One scientist (0.831%) in Multan reported spraying spinosad to control thrips on several crops.

### 3.5. Biological Control, Invasive Pathogen Dispersal, Quarantine, and SVNV-Infected Soy Feed Testing

**Figure 8:**
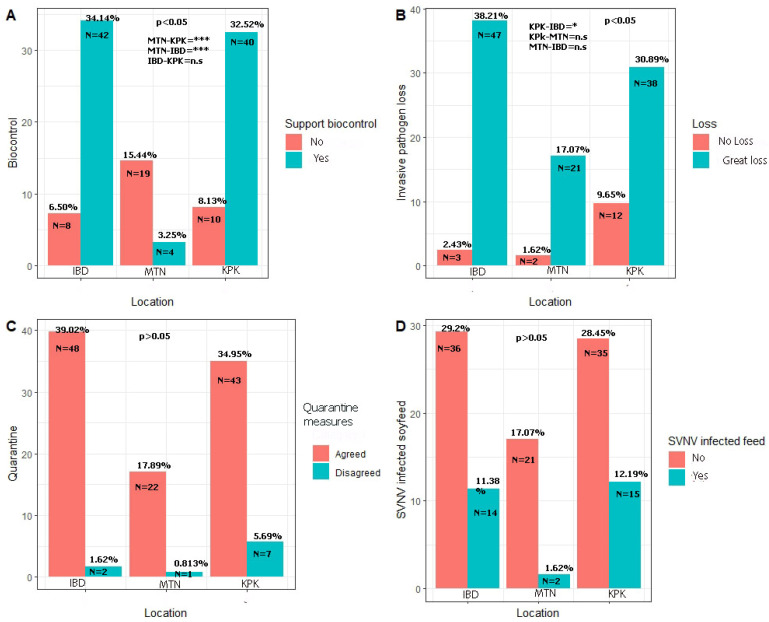
Farmers’ responses to different questionnaires. (**A**) Response regarding use of biocontrol agents in fields. (**B**) Response regarding invasive species knowledge. (**C**) Response regarding quarantine. (**D**) Response regarding SVNV-infected soymeal testing. Comparison is made through binomial modeling in R version 3.5.3. KPK, IBD, and MTN correspond to Khyber Pakhtunkhwa Province, Pakistan; Islamabad, Punjab, Pakistan; and Multan, Punjab, Pakistan. Here KPK-IBD means comparison of responses between farmers at location KPK and Islamabad. KPK-MTN means comparison of responses between farmers at location KPK and Multan. MTN-IBD means comparison of responses between scientists at location Multan and Islamabad. Here * represent significant difference below *p* < 0.05 while *** represents highly significant difference among mean responses at location IBD, MTN and KPK.

In response to biological control questions, significant differences were observed among the farmers’ and scientists’ responses at multiple locations (χ^2^ = 30.327, *p* < 0.01). Forty-two scientists (34.14%), forty farmers (32.52%), and four scientists (3.25%) in Islamabad, KPK, and Multan, respectively, reported that they like natural control organisms moving around soybean crops (Figure 8A).

When asked if an invasive-pathogen-infected seed would cause loss to the soybean crop, three scientists (2.43%), twelve farmers (9.75%), and two scientists (1.62%) in Islamabad, KPK, and Multan, respectively, reported that it would not cause a great loss (χ^2^ = 6.0361, *p* = 0.0489). However, 47 scientists (38.21), 38 farmers (30.89%), and 21 scientists (17.07%) in Islamabad, KPK, and Multan, respectively, reported that pathogen dispersal would cause a great loss (Figure 8B). Responding to a question about quarantine, most of the scientists and farmers agreed that the quarantine should be well-established to restrict the entry of invasive pests into Pakistan (*p* > 0.05) (Figure 8C). Two scientists (1.62%), seven farmers (5.69%), and one scientist (0.813%) in Islamabad, KPK, and Multan, respectively, reported that quarantine should not be established. Forty-eight scientists (39.02%), 43 farmers (34.95%), and 22 scientists (17.88%) in Islamabad, KPK, in Multan, respectively, replied that Pakistan should have well-developed quarantine measures to restrict the spread of pathogens in agriculture.

In response to the question of whether SVNV-infected soymeal should be given to livestock, 36 scientists (29.2%), 35 farmers (28.45%), and 21 scientists (17.07%) in Islamabad, KPK, and Multan, respectively, reported that it should not. However, 14 scientists (11.38%), 15 farmers (12.19%), and 2 scientists (1.62%) in Islamabad, KPK, and Multan, respectively, reported that SVNV-infected meal can be given to livestock (Figure 8D).

### 3.6. Reasons Behind Poor Adoption of Soybeans

**Figure 9:**
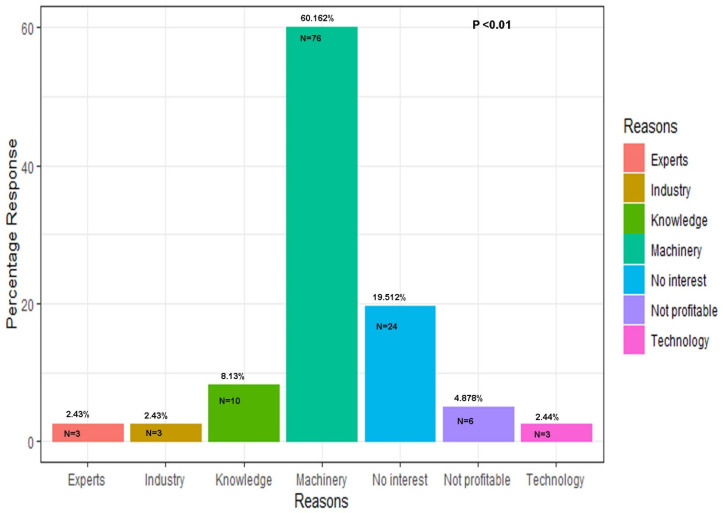
Farmers’ and scientists’ responses regarding reasons for poor adoption of soybeans in the farming community.

When asked why soybeans are not grown in Pakistan and what problems prevent extensive cultivation, most of the scientists and farmers selected lack of machinery, followed by technical knowledge, profit margin, few soybean experts, technology, and industry (*p* < 0.01, χ^2^ = 187.5) (Figure 9). Three respondents (2.43%) reported that the major problem is the lack of experts to advise farmers on how to cultivate soybeans in their fields (Figure 9). Three respondents (2.43%) reported that the major problem is that the industry does not buy the produce. Ten respondents (8.13%) reported that the soybean is not cultivated on their farms because they do not have the knowledge to grow soybeans. Seventy-six respondents (60.16%) reported that they do not grow soybeans because the machinery for harvesting and oil extraction is not available at a farmer’s scale (Figure 9). Twenty-four (19.512%) respondents reported that they do not grow soybeans because they do not have interest in this crop anymore (Figure 9). Six respondents (4.87%) reported that it is not profitable (Figure 9). Three respondents (2.433%) reported that the technology for planting, harvesting, and threshing is not available at the farmer’s doorstep (Figure 9).

### 3.7. Testing of Infected Seed, Insecticide Use, Resistant Cultivar Development, and Zero Tillage

When asked whether Pakistan should start testing for SVNV presence and produce cultivars which are resistant to SVNV (Table 2), about 80% of respondents at each location said that SVNV-resistant cultivars should be developed and a varietal screening process for SVNV should be initiated (*p* > 0.05) (Table 2). Asked whether insecticide should be used to control pests, a significantly higher number of scientists in Islamabad and farmers in KPK responded that insecticide use is the best option to control pests (*p* > 0.05, χ^2^ = 0.49) (Table 2). As for SVNV-resistant cultivar development, 98% of scientists (total 50) in Islamabad, 92% of farmers (total 50) in KPK, and 91% of scientists (total 23) in Multan replied that resistant varieties should be developed (Table 2).

### 3.8. Soybean Production Portfolio

**Figure 10:**
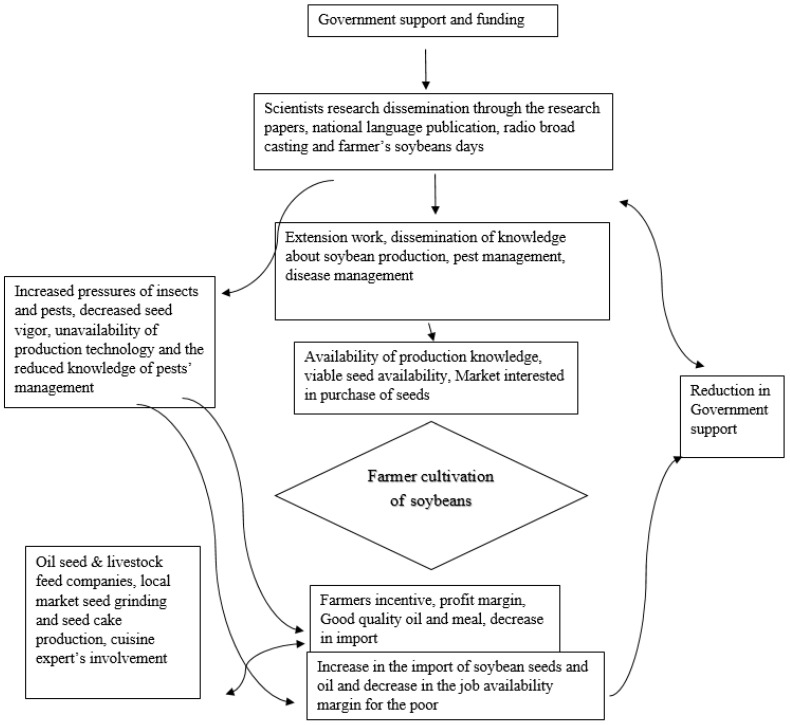
Soybean production flow chart, depicting factors which reduce soybean production and those that enhance the farmers’ profit.

A policy portfolio was developed that linked factors leading to poor adoption of soybeans in Pakistan. Overall, the decision to cultivate soybeans by farmers is linked with the marketability of soybeans. Government support and funding, including international support through the USDA, provided a chance for scientists to grow soybeans and develop soybean cultivars which were suitable to the agro-climatic condition of Pakistan. Scientists disseminate information through research papers, radio broadcasts, and farmer soybean days to help motivate the participating farmers to grow soybeans in their fields. Additional knowledge and support from extension officers may provide further incentive for farmers to grow soybeans. Overall, the availability of production knowledge, viable seed availability, expansion of the oil seed and seed cake market, and increased profitability will motivate farmers to grow soybeans. Increased profit margins for farmers may help to boost soybean production, enhancing the gross domestic production and decreasing the import of soy products. On the other hand, decreased government support for scientists and extension officers, including the private sector’s purchase of seeds from the international markets, would have a negative effect on soy production in Pakistan (Figure 10).

### 3.9. Soybean-Vein-Necrosis-Infected Plants in Scientists’ Laboratories

Photographs of the plants infected with soybean vein necrosis were developed, and videos taken, in scientists’ places of study (Figure 2). The photographs showed necrotic leaves, demonstrating typical symptoms of SVNV.

## 4. Discussion

We determined SVNV presence as well as awareness of soybean production and disease management in the Pakistani community, specifically asking questions about SVNV, its vectors, and the reasons for poor adoption of soybean production in Pakistan, in a survey of both scientists and farmers. We conclude that the problem of low soybean cultivation in Pakistan is complicated. Soybean production requires the efficient transfer of information from scientists to farmers and an enhanced government role to incentivize the industry. Farmers need an established local industry to generate additional income by selling the crop to seed oil extraction and feed firms (Figure 10).

Overall, the number of soybean scientists is limited in Pakistan; only a few scientists (including two graduate students) were found in southern Punjab, while thirteen soybean scientists were located in the capital city of Islamabad. Overall, the knowledge of soybean crop varieties and crop production technologies was higher among scientists in Islamabad. One of the reasons for this may be that the NARC in Islamabad took some initial steps to introduce soybeans in Pakistan, importing soybean cultivars from Ohio State University and growing soybeans on a pilot scale at the institute; hence, these scientists are more familiar with soybeans than those in Multan, which is in the high-temperature zone of Pakistan and well known for cotton and mangoes [31,34,35]. Multan being the hub of cotton production, most scientists there work only on cotton. Unfamiliarity with the soybean crop may be due to this specialization on other crops (mangoes and cotton).

The problem seems to be that the extension education program provided to farmers and the overall agricultural community was not effectively developed, and thus, overall knowledge about soybean cultivation did not become common in southern Punjab. Furthermore, the Multan part of southern Punjab is the cotton-growing area and Pakistan is the top exporter of the cotton, so a lack of knowledge of soybean cultivars may be due to a lack of knowledge about soybean production technology. Most soybeans are cultivated in Islamabad on 150 acres at the NARC research institute, but in Multan, we were not able to find even a single soybean farm. Having observed the soybean production process, the scientists at the NARC in Islamabad were aware of soybean production technologies even if they were not working on soybeans.

It was interesting to observe that most of the soybean farmers were present in KPK Province. KPK is closer to the NARC in Islamabad, which is the main soybean variety development research institute in Pakistan, while the southern Punjab city of Multan is farther away. Landholding capacity of Pakistani farmers was low. Most farmers had 0.40–4.04 ha of land and were primary-qualified. In the United States, a small farmer is one with less than 400 ha, compared to which these farmers have very little. They grow soybeans in units called kanals (each one having an area about eight times less than one acre), and this soybean is used for livestock feed.

Most farmers, on observing the SVNV symptoms pictured on the survey questionnaire, said that they have seen similar symptoms in their fields. The farmers were not aware of the name of disease, but after observing symptomatic leaves in color photographs, they said that they had seen in their fields the necrotic vein and purple blotched leaves that are characteristic symptoms of SVNV.

In multiple-choice questions about vector thrips species, most of the scientists in Islamabad selected *Frankliniella occidentalis* as the vector of SVNV. This might be because of increased knowledge available about the association between TSWV and *F. occidentalis* in the Pakistani agro-ecosystem. We still could not find any report or thesis describing the presence of *F. occidentalis* in Pakistani agricultural fields. Most of the scientists selected were not able to confirm the vector species among the list of the species mentioned. This means that although SVNV has been recognized since 2008 in the USA, Pakistani scientists’ general knowledge about SVNV has not been developed yet.

In Pakistan, the portfolio for the pest management of soybeans relies on the use of pesticides. However, globally, different approaches are used for pest management, such as the use of insecticide- and fungicide-treated seed, zero tillage, inoculation with *Rhizobium* bacteria (*Bradyrhizobium japonicum*), *Cucumeris* mites and *Orius insidiosus* card installation, novel insecticides, application of glyphosate-resistant seed, storage of seed at low temperature (4 °C), and phyto-hormone applications [36,37,38,39,40]. In response to questions about biological control, farmers and scientists in Pakistan indicated a preference for the natural control of pests, but natural control is dependent upon maintaining biological control fauna at higher numbers, which seems to be impossible due to high pesticide use [41,42,43,44]. Further, in Pakistan, no agency supplies the biological agents to control the pests, so farmers and scientists rely on locally occurring natural control agents, which are decreasing in abundance and diversity because of pesticide use.

Research institutes of the Pakistani government are the only agencies which provide seeds, and insecticide-treated seeds from the government are not available. Farmers themselves, if they are knowledgeable, apply a seed treatment; otherwise, the seed treatment with fungicide and insecticide is not practiced. No cover crop or shelter crop is planted for biocontrol agents to survive and predate on pests, but intercropping is practiced with mung beans, mash beans, and cotton [33]. Mung bean is an alternate host of SVNV, and cotton is a highly attractive crop to whiteflies, another important vector of plant viruses [16,45]. Thus, the pests continue to thrive on the crop and build their populations earlier in the season, while in the U.S., the pest (thrips) attack occurs later in the season. This early build-up of higher pest inoculum in Pakistan leads to early viral disease symptoms, appearing even one month after sowing, while in the northern states of the USA, SVNV symptoms appear 2 months after sowing. Hence, the soybean crop in the northern U.S. escapes the disease’s worst intensity due to reduction in pest numbers and utilization of crop protection technologies, including the sowing of insecticide-treated seeds and herbicide-resistant seeds. Moreover, the pest migratory and overwintering pattern in the northern soybean-growing states reduces pest survival during migration and follow-up survival [46]. In addition, the northern states in the U.S. have a high rainfall index, which reduces the population build-up of thrips to a great extent [47]. In Punjab, Pakistan, there is a large area with saline soils [48], on which soybeans can be grown along with some cover crops. However, the soybean production portfolio advertised by the government does not inform farmers that they can plant soybeans on the saline soil. Saline soil farmers in Pakistan grow mostly *Dactyloctenium aegyptium* as forage. The planting of soybeans in saline soil can increase the soybean production in Pakistan overall, and the farmers of Punjab would obtain better forage for their livestock, enhancing milk and cattle production.

The decision to plant a crop starts from economic considerations, including the profit margin, the presence of a market for the farmers to sell the products, and a government or regulating agency incentive for farmers and the industry for commercialization of the product. The low production of soybeans does not stimulate the market development that would enable the industry to buy soy seeds directly from farmers and Pakistani indigenous markets. Hence, the industry buys the produce from other countries in bulk, and then processes the seed into soymeal and seed cake [29,33,49]. In Pakistan, Eid-ul-Adha is a significant holistic religious event. Farmers in Pakistan grow animals and sell them during this time at good prices [49]. Animals are rated on the basis of their health and height. In order to obtain the maximum profit, farmers buy soymeal cake from the market to improve the health of their heifers and bulls, which increases the cost of animal production and decreases the farmers’ profit. If the production technology of soybeans is extensively propagated in the farming community of Pakistan through the work of scientists and researchers, the farmers may not need to buy the meal cake from the market, hence their profit margin can be increased. Furthermore, if farmers cultivate soybeans on a large scale, then the industry will buy from them, and thus the farmers’ (70% of the population of Pakistan) living standards can be improved. There is a need is to restructure the soybean production plan in Pakistan. A close interaction among government support, farmer NGOs, soybean scientists, soybean extension agents, and farmers is needed to increase soybean production in Pakistan.

In Pakistan, the problems of soybean cultivation are numerous. For example, after soybean cultivation, seedling pests called thrips attack the crop, after which whiteflies and armyworms become dominant pests. Farmers spray mainly to kill the whiteflies and armyworms; however, it is not known whether whiteflies transmit any kind of virus in soybeans. It should be considered that in Punjab (a fertile region of Pakistan), cotton is the main crop, which is attacked by whiteflies (vector of cotton leaf curl virus). However, the presence of replicating CLCuV in soybeans has not been reported yet. The question of an increase in herbivory in virus-infected plants has been addressed by research, which showed that cross-talk between the two defense pathways against herbivory and viruses results in an increase of herbivores. Eventually, the best competitor is one that can increase numbers quickly. This is also evident in Pakistani soybean cultivation. We found that the fields of soybeans showing SVNV symptoms were also filled with whiteflies. We postulate that cross-talk between the SVNV and Jasmonic acid enhances the spread of whiteflies on soybean fauna in Pakistan.

Biocontrol agents play an important role in reducing pest numbers. Agriculture in the Indus valley is ancient. Pakistan is rich in biocontrol agents, which decrease pest numbers. The problem is that augmentation and conservation of these biocontrol agents on a large scale is difficult. It is necessary to educate farmers in developing shelter belts, identifying biocontrol agents, and counting the biocontrol agents before spraying to reduce the spray’s harmful effects. Farmers introduced to a biocontrol appraisal program can act as role models for the farming community.

Overall, an integration of these efforts is needed on the part of both farmers and scientists to reduce the spread of pests and enhance farmers’ income.

## 5. Conclusions

This research represents the first novel study linking farmers’ and scientists’ perceptions of soybean vein necrosis orthotospovirus and thrips to overall soybean adoption by the farming community in Pakistan. Overall, we reported the following. (1) Although scientists were aware of thrips, they did not recognize them as vectors of SVNV and were not familiar with SVNV disease. (2) The import of a new crop to a new country should be accompanied by knowledge of its diseases and vectors before spreading the contaminated seeds to the farming community. (3) With this study, we can say that SVNV is present in the USA, Canada, Egypt, and Pakistan. (4) Pakistan is a developing country with less economic resources, requiring a deeply rooted agricultural policy portfolio, which should include the knowledge of pest management, crop production options, and production technology information including the machinery for harvesting soybean meal and soybean oil extraction, to maximize soybean production in Pakistan. (5) Although scientists and farmers intend to use less insecticide, and would prefer to use biocontrol agents, extensive education is needed to train them about local biocontrol fauna and local practices that farmers can adopt to increase profits and decrease the abundance of pests and diseases. (6) Improving soybean production will help the farming community and common consumers and increase farmers’ overall profit.

## Figures and Tables

**Table 1:** Scientists involved in different crop research.

Location	Crop	Scientists
Multan	Cotton	12
Multan	Soybeans	2
Multan	Mango	8
Multan	Pomegranate	1
Islamabad	Pomegranate	1
Islamabad	Wheat	15
Islamabad	Soybeans	21
Islamabad	Rice	4
Islamabad	Sunflower	1
Islamabad	Safflower	1
Islamabad	Peanut	7

**Table 2:** Scientists’ and farmers’ responses regarding the testing of seeds for SVNV before variety development, insecticide use to control thrips, SVNV-resistant cultivar development, and zero tillage as an option to enhance biocontrol in Pakistani soils.

Question	Response	Ibd %	KPK %	Mtn %	*p*-Value	Chi Value
Testing of imported seed before varietal development	No	1.62	6.50	2.43	0.19	3.24
	Yes	39.02	34.14	16.26		
Insecticide use	No	1.62	2.43	2.43	0.416	1.751
	Yes	39.02	38.21	16.26		
Resistant cultivar development	No	0.813	3.252	1.626	0.416	1.75
	Yes	39.837	37.389	17.073		
Zero tillage	No	6.50	5.69	4.87	0.5023	1.377
	Yes	34.14	34.95	13.82		

## Data Availability

All data related to this study is contained within the paper.

## Supplementary Materials

The following supporting information can be downloaded at: https://www.mdpi.com/article/10.3390/v17030315/s1. Table S1: List of questions asked of scientists and farmers regarding SVNV presence, dispersal, and future management through adopting the selective breeding program; Table S2: Presence of SVNV by qRT-PCR in plants and thrips in Pakistan through nucleocapsid genes (NP) and silencing suppressor genes (NSS). Table S3: A summary of the number of plants tested through ELISA for each cultivar and disease incidence percentage.
