# Supplementary material for: Presence of Soybean Vein Necrosis Orthotospovirus (Tospoviridae: Orthotospovirus) in Pakistan, Pakistani Scientists’ and Farmers’ Perception of Disease Dynamics and Management, and Policy Recommendations to Improve Soybean Production
Source: Viruses. 2025 Feb 25;17(3):315. doi: 10.3390/v17030315 (PMC11946773; doi:10.3390/v17030315)
Supplement: Supplementary file 1 [file viruses-17-00315-s001.zip › viruses-3389633-supplementary.pdf]

Table S1. List of questions asked of scientists and farmers regarding SVNV presence, dispersal, and future management through adopting the selective breeding program.

Sr.	Scientists	Farmers
**1.**	On what crop are you conducting your research experiments?	How many acres of land do you have?
**2.**	What kind of soybean cultivar have you grown on your research farm?	
**3.**	Do you know about SVNV, a new seed-borne virus in the USA?	What level of education do you have?
**4.**	Common questions to both groups	
**5.**	Have you seen SVNV-symptomatic plants in your research field or farm fields?	
**6.**	Do you know insects? Which insect is thrips among these?	
**7.**	Can you recognize which thrips species is a vector?	
**8.**	Do you spray your field to control thrips?	
**9.**	What kind of insecticide do you use on your field?	
**10.**	Do you use biological control agents to control the thrips population?	
**11.**	Do you think that, if insects/seed-borne viruses which cause disease in soybean come along with imported commodities including flowers and seed, it would cause loss to the soybean crop?	
**12.**	Do you think that the Government of Pakistan should have quarantine measures well established to restrict entry of invasive pests and diseases?	
**13.**	Do you think that the livestock feed made from seed infected with SVNV should be given to livestock?	
**14.**	In Pakistan, varieties are developed through crossing with seeds imported from developed countries. Do you think that these imported seeds should be cultivated without checking for disease presence?	
**15.**	Why are soybeans not grown in Pakistan? What are the problems which don't allow extensive cultivation?	
**16.**	Do you think that insecticides should be used to control thrips?	
**17.**	Do you think that virus-resistant cultivars should be developed and that Pakistan should improve its research institutes?	
**18.**	Do you know about zero-tillage programs? Do you think that if Pakistan adopted zero-tillage, then we could encourage the use of biological control agents?	

Table S2. Presence of SVNV by qRT-PCR in plants and thrips in Pakistan through nucleocapsid genes (NP) and silencing suppressor genes (NSS).

**Dated 26 August 2019**			
**Sr.**	**Treatment**	**Gene**	**Avg.CT**
1	Water	NSS	NaN
2	Water	NSS	NaN
3	Water	NP	NaN
4	Water	NP	NaN
5	Water	GM	NaN
6	Water	GM	NaN
1	BT	NP	12.10746
2	BT	NP	11.41231
3	BT	NP	10.83541
1	BT	NSS	24.15024
2	BT	NSS	26.83416
3	BT	NSS	25.81014

1	HP	NP	NaN
2	HP	NP	NaN
3	HP	NP	NaN
1	HP	NSS	NaN
2	HP	NSS	NaN
3	HP	NSS	NaN
1	IP	NP	15.43211
2	IP	NP	14.83214
3	IP	NP	15.12916
1	IP	NSS	37.92724
2	IP	NSS	35.42135
3	IP	NSS	36.21431
1	HP	GM	26.43116
2	HP	GM	27.81465
3	HP	GM	28.91425
1	IP	GM	26.05838
2	IP	GM	27.15242
3	IP	GM	25.61251

Dated 13 September 2019

**Sr.**	**Treatment**	**Gene**	**Avg.CT**
1	Water	NSS	NaN
2	Water	NSS	NaN
3	Water	NP	NaN
4	Water	NP	NaN
5	Water	GM	NaN
6	Water	GM	NaN
1	BT	NP	13.67054
2	BT	NP	15.12394
3	BT	NP	11.83211
1	BT	NSS	25.80024
2	BT	NSS	28.07713
3	BT	NSS	30.81334
1	HP	NP	NaN
2	HP	NP	NaN
3	HP	NP	NaN
1	HP	NSS	NaN
2	HP	NSS	NaN
3	HP	NSS	NaN
1	IP	NP	17.47098
2	IP	NP	18.22865
3	IP	NP	18.53194
1	IP	NSS	25.13333
2	IP	NSS	25.89041
3	IP	NSS	26.31421
1	HP	GM	23.25051
2	HP	GM	21.28416
3	HP	GM	24.51342

1	IP	GM	24.90104
2	IP	GM	23.21230
3	IP	GM	24.83514
**Dated: 20 September 2019**			
**Sr.**	**Treatment**	**Gene**	**Avg.CT**
1	Water	NSS	NaN
2	Water	NSS	NaN
3	Water	NP	NaN
4	Water	NP	NaN
5	Water	GM	NaN
6	Water	GM	NaN
1	BT	NP	24.75192
2	BT	NP	25.80498
3	BT	NP	25.75821
1	HP	NP	NaN
2	HP	NP	NaN
3	HP	NP	NaN
1	HP	NSS	NaN
2	HP	NSS	NaN
3	HP	NSS	NaN
1	IP	NP	14.18448
2	IP	NP	12.33514
3	IP	NP	14.76059
1	IP	NSS	26.73
2	IP	NSS	26.721
3	IP	NSS	21.568
1	HP	GM	30.76
2	HP	GM	30.8
3	HP	GM	31.23
1	IP	GM	30.76
2	IP	GM	30.23
3	IP	GM	29.82
**Dated: 26 September 2019**			
**Sr.**	**Treatment**	**Gene**	**Avg.CT**
1	Water	NSS	NaN
2	Water	NSS	NaN
3	Water	NP	NaN
4	Water	NP	NaN
5	Water	GM	NaN
6	Water	GM	NaN
1	BT	NP	25.80498
2	BT	NP	26.41323
3	BT	NP	22.12451
1	BT	NSS	25.73655
2	BT	NSS	10.40679
3	BT	NSS	25.81234
1	HP	NP	NaN

2	HP	NP	NaN
3	HP	NP	NaN
1	HP	NSS	NaN
2	HP	NSS	NaN
3	HP	NSS	NaN
1	IP	NP	12.66134
2	IP	NP	14.18448
3	IP	NP	11.33514
1	IP	NSS	26.79124
2	IP	NSS	26.73218
3	IP	NSS	26.72193
1	HP	GM	30.81213
2	HP	GM	31.42153
3	HP	GM	29.52145
1	IP	GM	29.80893
2	IP	GM	30.41235
3	IP	GM	29.51432

BT = black thrips, a common thrips species found in soybean crops in Pakistan. HP = asymptomatic plant. IP = symptomatic plant. NP = nucleocapsid gene. NSS = silencing suppressor gene. GM = *Glycine max* internal control factor. GOI = gene of interest.

Table S3. A summary of the number of plants tested through ELISA for each cultivar and disease incidence percentage.

Variety	Number of Plants Tested	Number of Plants Positive Through ELISA	Incidence Percentage
Ajmeri	20	20	100
Williams 98	20	4	20
Rawal-1	20	9	45
NARC-2	20	12	60
SA7260	20	13	65
Faisal P-17	20	20	100
NARC-16	20	2	10
